# Three years of pulmonary rehabilitation: inhibit the decline in airflow obstruction, improves exercise endurance time, and body-mass index, in chronic obstructive pulmonary disease

**DOI:** 10.1186/1471-2466-9-26

**Published:** 2009-05-30

**Authors:** David Stav, Meir Raz, Isaac Shpirer

**Affiliations:** 1 Pulmonary Institute, Tel Aviv University, Assaf Harofeh Medical Center, Zerrifin, Israel; 2 Tel Aviv Lung Association, Tel Aviv, Israel; 3 Maccabi Health Services, Tel Aviv, Israel

## Abstract

**Background:**

Pulmonary rehabilitation is known to be a beneficial treatment for COPD patients. To date, however, there is no agreement for how long a rehabilitation program should be implemented. In addition, current views are that pulmonary rehabilitation does not improve FEV_1 _or even slow its decline in COPD patients. The aim of the study was to examine the efficacy of a 3 year outpatient pulmonary rehabilitation (PR) program for COPD patients on pulmonary function, exercise capability, and body mass index (BMI).

**Methods:**

A matched controlled trial was performed with outcome assessments evaluated at 6, 12, 18, 24, 30, and 36 months. Eighty patients with moderate to severe COPD (age 63 ± 7 years; FEV_1 _48% ± 14) were recruited. The control group received standard care only, while in addition, the case study group received PR for duration of three years. These groups were matched for age, sex, BMI, FEV_1_% and number of pack-years smoked.

**Results:**

The decline in FEV_1 _after the three years was significantly lower in the PR group compared to control, 74 ml versus 149 ml, respectively (p < 0.001). Maximal sustained work and endurance time improved after a short period of PR and was maintained throughout the study, in contrast to the control group (p < 0.01). A decreased BMI was noted in the control group after three years, while in the PR group a mild improvement was seen (p < 0.05).

**Conclusion:**

Three years of outpatient pulmonary rehabilitation resulted in modifying the disease progression of COPD, as well as improving physical performance in these patients.

## Background

Chronic obstructive pulmonary disease (COPD) is a chronic progressive respiratory disorder causing disability with an increasing burden to the patient, his family and to the health services. Ppatients with COPD commonly experience reduced exercise capacity and activity limitation. Furthermore, exercise intolerance is a major determinant of impaired quality of life for COPD patients, and improvement in exercise capacity is a key goal of COPD disease management [1].

Drug treatment regimens provides only partial benefit, and many patients remain symptomatic with impaired exercise capacity and and a worsening quality of life. For example, prospective studies testing for the effects of inhaled short-acting anticholinergic drugs, inhaled corticosteroids, or *N*-acetylcysteine on the progression of chronic obstructive pulmonary disease (COPD) failed to demonstrate a change in the slope of the forced expiratory volume in 1 second (FEV_1_) in these patients [2-4]. To date, only smoking cessation has prospectively been shown to alter the rate of decline of FEV_1 _in patients with COPD [5]. Pulmonary rehabilitation (PR) is used as a complementary treatment option for these patients, Recently, the American Thoracic Society (ATS) and the European Thoracic Society (ERS) published a statement, in which PR was recognized as an evidence-based, multidisciplinary, and comprehensive intervention for patients with chronic respiratory diseases who are symptomatic and often have decreased daily life activities. [6]. This statement was followed by a position paper by the ACCP/AACVPR that support and enhance the previously described statement [7]. One of the major unresolved issues is the duration of treatment. For example, outpatient exercise training with two or three weekly sessions for 4 weeks showed less benefit than similar training for 7 weeks [8-10].

In order to evaluate the efficacy of pulmonary rehabilitation to alter the course of disease in COPD, we carried out in an outpatient setting, a controlled trial comparing 36 months of PR along with standard care and to the effects of standard care without rehabilitation. The outcome measurements included changes in the rate of FEV_1 _decline, its effects on exercise endurance and the change of body mass index (BMI).

## Methods

COPD patients who were on long acting beta agonist (LABA) or combined inhalers of corticosteroids and LABA, were matched for age, sex, BMI, FEV_1_% and number of pack-years smoked into two groups, those receiving pulmonary rehabilitation in addition to standard care and a control group receiving inhaled drugs as described above [11]. Patients were eligible for inclusion if they were younger than 70 years of age, had a FEV_1 _that was less than 60% and higher than 30%, of predicted value and their improvement after bronchodilator inhalation (400 μg salbutamol) was less than 12%. In addition, their clinical condition had been stable for at least 2 months prior to enrollment. We excluded patients who were active smokers or had quit smoking less than 2 years prior to the onset of this study and those in whom there were other severe medical problems such as heart failure, myocardial infarction, cerebrovascular disease, cancer, or severe orthopedic disorders.

PR was carried out in groups of 6–8 patients, twice a week during the course of the study. Each session consisted of exercises of both upper and lower extremities as well as integrated physical activity. This program was directed by an exercise therapist. Patients were instructed to carry out exercises at home simulating what they did in the groups, on at least two additional days per week. In addition, meetings on an individual basis with a psychologist, sometimes with family members included, occurred as needed. The patients were seen initially by the supervising physician and at intervals of three months for routine follow up and for functional evaluations. In addition, physician encounters occurred as needed. The control group was similarly seen and assessed by a pulmonary physician at three months intervals, in addition to as needed.

Pulmonary function studies were carried out at enrollment and at intervals throughout the study period. Spirometry (Medical Graphics, Inc., St. Paul, Minnesota) including FEV_1 _and forced vital capacity, were measured according to American Thoracic Society guidelines [12]. The BMI was re-calculated at every spirometry.

### Exercise Endurance

During the run-in period, exercise tolerance was evaluated with a constant-load cycle ergometer on two separate visits. The constant-load tests were performed at 75% of the maximal work rate achieved during the incremental exercise test at screening. The purpose of the run-in exercise tests was to familiarize the study subjects with the constant-load exercise test procedures and reduce possible learning effects [13]. Cycle endurance time was defined as the duration of loaded pedaling. For the incremental test, the initial work rate was 10 Watts (W) and the work rate was increased by 10 W every minute until symptom limitation. Pulse oximetry, ECG, and BP were monitored at rest, during exercise, and at recovery.

### Statistical Analysis

Data are presented as mean ± SD. Analysis of variance (ANOVA) was used to compare between the groups for analysis of the entire 36 months period. For time point differences we used, a two-sample t test with a significance level of 0.05.

### Study ethics

The study was approved by the Tel Aviv Lung Association, Institutional Review Board

 – TLA1948

## Results

Eighty consecutive eligible patients fulfilled inclusion criteria and were matched to either the training group (40) or the control group (40). Thirteen patients, 16% (6 in the rehabilitation group and 7 in the control group) did not complete the 3 years of follow up (Table 1). All patients had moderate-severe COPD with moderate peripheral and respiratory muscle weakness and impaired functional and maximal exercise capacity. There were no significant differences in the characteristics of the 80 patients who initially participated in the PR and control groups (Table 1).

**Table 1:** Base line characteristics of the study subjects

	Control group			Rehabilitation group		
	
	Completed	Withdrawn	All	Completed	Withdrawn	All
No.	33	7	40	34	6	40
Age	62 ± 5.4	64 ± 2.8	63.2 ± 5	63 ± 5.2	63.2	62.6 ± 6
Male	70%	76%	72%	72%	75%	75%
BMI	24.6 ± 1.8	24.2 ± 1.4	24 ± 1.6	24.2 ± 1.9	23.8 ± 1.6	24.1 ± 1.7
FEV_1_%	44 ± 8	46 ± 7	44 ± 9	47 ± 5	48 ± 6	47 ± 8
FEV_1_/FVC	0.56 ± 0.06	0.54 ± 0.04	0.52 ± 0.07	0.56 ± 0.08	0.52 ± 0.03	0.54 ± 0.06
Pack year	24 ± 7	27 ± 4	26 ± 8	27 ± 6	30 ± 4	28 ± 10

BMI-body mass index, FEV_1_%-percent of predicted of forced expiratory volume in 1 second

**Figure 1:**
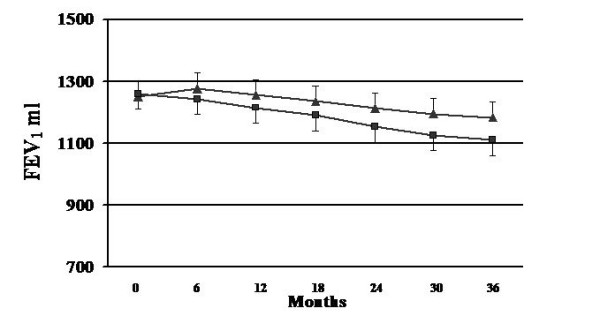
**Evolution of FEV_1 _decline, rehabilitation group represented by line and black triangles, whereas control group by line and black squares**. A significant difference in the rate of FEV_1 _over time is noted between these two groups (p < 0.001).

The rate of FEV_1 _decline was measured after administration of a 400 ug of salbutamol, a short acting bronchodilator. Figure 1 depicts the changes in FEV_1 _over time in the pulmonary rehabilitation and control groups, and demonstrates a significant difference (p < 0.001) between these two groups. In addition, the decline of FEV_1 _in the PR group was significantly lower when comparing each individual time point (p < 0.05). In the PR group the FEV_1 _was reduced by 74 ml during the 3 years study, whereas in the control group it was 149 ml. Table 2 demonstrates an immediate significant improvement (p < 0.001) in exercise endurance time and work in the PR group, which was basically maintained during the study duration. In contrast, in the control group no significant differences was observed. BMI improvement in PR treated group was significant after more than 1 year, and an opposite change was observed in the control group.

**Table 2:** Effect of pulmonary rehabilitation on cycle exercise performance and BMI

	Control group			Rehabilitation group			
	Before	After 1-year	After 3-years	Before	After 1-year	After 3 years	P-value
Max. sustained cycle work, W	32 ± 11	30 ± 8	28 ± 8	30 ± 9	76 ± 22	78 ± 24	< 0.01
Max. sustained cycle time, Min.	5.8 ± 0.9	5.6 ± 1.1	5.8 ± 1.1	6 ± 1.1	12.6 ± 4.4	11.8 ± 4	< 0.01
BMI	24.6 ± 1.8	24.4 ± 2.2	21.6 ± 2.4*	24.2 ± 1.9	25.4 ± 2	25.1 ± 2.2	< 0.05

Max. -maximum, W-watts, min.-minutes, *- only 3 years was significant, BMI-body mass index

## Discussion

Our study demonstrates that a prolonged rehabilitation program inhibits the progression of airflow obstruction in COPD patients. In addition, it increased endurance time and work, and improved BMI. FEV_1 _is used in COPD for disease staging and since it declines progressively, its value is a predictor of life expectancy. Various exercise tests, different physiological parameters, and BMI are used as surrogates for clinical status assessment. Our study did not show that PR improved FEV_1_, which is in accordance to a previous report, but showed beneficial interference with its progressive decline.

Previous studies have shown some improvement in FVC which may have been due to improved respiratory muscle function and a reduction in small airways disease [3]. The improvement in FEV_1 _in those cases was small and not statistically significant. We noted a significant inhibtion of the progression of airways obstruction occurring after the three years of treatment, and thus we speculate that participating in the pulmonary rehabilitation program increased in incremental stages small airways function and/or recruitment. In addition, the exercise regimen likely improve secretions evacuation, which can reduce airways infections/inflammation and decrease COPD exacerbations. For example, it was recently shown that moderate to high levels of regular physical activity are associated with a reduced lung function decline and COPD risk among smokers [14]. Furthermore, in earlier reports regular exercise was noted to protect against diseases associated with chronic inflammation [15]. Inflammation is an important element in the pathogenesis of COPD. The contribution of PR for reduction of FEV_1 _decline adds an additional beneficial effect of pulmonary rehabilitation for COPD patients. FEV_1_decline may serve as a predictor of risk death from COPD, and therefore PR should be considered as a disease modifier [16]. The improvement of endurance time and work noted in the trial is consistent with previous reports [17]. One possibility no significant differences were noted regarding exacerbation-related hospitalizations in these two groups, may relate to the higher accessibility to medical care in the patients enrolled in our study, relative to the regular COPD patient. A randomized study would be preferable, in

Match study the control subjects who are 'matched' with the treated subjects on background covariates that the investigator believes need to be controlled [11]. Finally, since this study included patients with severe disease, the duration of three years to complete the study may have contributed to the relatively high number of dropouts (including death), for which we could not identify a specific reason.

## Conclusion

Three years of pulmonary rehabilitation has an important beneficial impact on the rate of FEV_1 _decline, in addition to previously reported advantages of this treatment modality, for COPD patients.

## Competing interests

The authors declare that they have no competing interests.

## Authors' contributions

DS design the study, was responsible to the rehabilitation group and data analysis, and manuscript writing. MR participated in its design, data acquisition, coordination and drafted the manuscript IS was responsible to the matched control group, data acquisition and analysis, and reviewing manuscript.

## Pre-publication history

The pre-publication history for this paper can be accessed here:


